# MicroRNA categorization using sequence motifs and k-mers

**DOI:** 10.1186/s12859-017-1584-1

**Published:** 2017-03-14

**Authors:** Malik Yousef, Waleed Khalifa, İlhan Erkin Acar, Jens Allmer

**Affiliations:** 1 Community Information Systems, Zefat Academic College, Zefat, 13206 Israel; 2 Computer Science, The College of Sakhnin, Sakhnin, 30810 Israel; 3 Biotechnology, Izmir Institute of Technology, 35430 Urla, Izmir Turkey; 4 Molecular Biology and Genetics, Izmir Institute of Technology, 35430 Urla, Izmir Turkey; 5 Bionia Incorporated, IZTEKGEB A8, 35430 Urla, Izmir Turkey

**Keywords:** microRNA, Sequence motifs, Pre-microRNA, Machine learning, Differentiate miRNAs among species, k-mer, miRNA categorization

## Abstract

**Background:**

Post-transcriptional gene dysregulation can be a hallmark of diseases like cancer and microRNAs (miRNAs) play a key role in the modulation of translation efficiency. Known pre-miRNAs are listed in miRBase, and they have been discovered in a variety of organisms ranging from viruses and microbes to eukaryotic organisms. The computational detection of pre-miRNAs is of great interest, and such approaches usually employ machine learning to discriminate between miRNAs and other sequences. Many features have been proposed describing pre-miRNAs, and we have previously introduced the use of sequence motifs and k-mers as useful ones. There have been reports of xeno-miRNAs detected via next generation sequencing. However, they may be contaminations and to aid that important decision-making process, we aimed to establish a means to differentiate pre-miRNAs from different species.

**Results:**

To achieve distinction into species, we used one species’ pre-miRNAs as the positive and another species’ pre-miRNAs as the negative training and test data for the establishment of machine learned models based on sequence motifs and *k*-mers as features. This approach resulted in higher accuracy values between distantly related species while species with closer relation produced lower accuracy values.

**Conclusions:**

We were able to differentiate among species with increasing success when the evolutionary distance increases. This conclusion is supported by previous reports of fast evolutionary changes in miRNAs since even in relatively closely related species a fairly good discrimination was possible.

**Electronic supplementary material:**

The online version of this article (doi:10.1186/s12859-017-1584-1) contains supplementary material, which is available to authorized users.

## Background

Gene expression can be fine-tuned on several levels, but dysregulation often leads to disease. MicroRNAs (miRNAs) are involved in post-transcriptional gene regulation [1] which modulates protein abundance by fine-tuning translation rates [2]. MicroRNAs contain a short stretch of nucleotides (~20) acting as a recognition sequence to direct the RNA-induced silencing complex (RISC) complex to its target mRNA. This regulation mechanism exists in a wide range of species like viruses [3] and plants [4]. Although the plant miRNA pathway is said to have evolved independently of the metazoan one [5], the secondary pre-miRNA structures appear to be similar when visually inspected on miRBase [6] which houses known pre-miRNAs and their mature miRNAs. Release 21 of miRBase contains 28,645 mature miRNAs (2588 for human), but the existence of many more miRNAs can be expected [7]. The experimental detection of miRNAs is, however, convoluted by the fact that they can only convey function when co-expressed with their target mRNAs [8]. Therefore, and since it seems futile to try and discover all miRNAs of an organism experimentally, computational prediction of miRNAs has become important. Most such approaches employ machine learning using two-class classification [9, 10].

The so-called ab initio miRNA detection methodology has been well established in animals [11], and we have shown that it also works well in plants [4]. Machine learning depends on the parameterization of the biological structure, and many features have been described to represent a pre-miRNA numerically [12, 13] to which we have recently added sequence motifs [14]. These features are used to differentiate between the positive (miRNA) and the negative class employing a variety of classifiers like support vector machines [15] and random forest [16]. Unfortunately, bona fide negative pre-miRNA examples do not exist and, therefore, using two-class classification is limited and suffers from the use of arbitrary negative data of unknown quality [17].

Here we used similar strategies as other two-class classification approaches for pre-miRNA detection, however, with a different intention. The purpose of the present study was to differentiate pre-miRNAs between two species. That means both positive and negative classes for training were derived from known pre-miRNAs which removed the need to employ pseudo negative data. This approach is viable for miRNAs because fast evolution has been shown to exist for them before [18–20] so that given larger evolutionary distances at least the miRNA sequences should deviate enough to allow discrimination. Hence, we focused on sequence-based features and motifs to achieve proper discrimination. Previously, Ding et al. used n-grams (same as our k-mers) to create miRNA families [21], which was a similar intention but from a different perspective. Ding et al. tried to solve the multi-class problem of assigning an unknown miRNA to its correct miRNA family which does not represent a species but the membership of a miRNA to a family of miRNAs which consists of miRNAs from different species, which are evolutionary conserved. Lopes et al. also attempted to discriminate between species [22], but used the same synthetic negative data that is generally used in pre-miRNA detection methods [23–26] and employed the same training and testing strategies as other approaches [16, 27–29]. They further focused on structural features which we found not to be useful for discriminating between closely related species since the structure is generally more conserved than sequence composition. An important contribution of the present work is that it overcomes the use of arbitrary negative examples of unknown quality by using the data of one species for positive examples and the data of the other species for negative examples and vice versa. In summary, one of the purposes of the present study was to discriminate between two species using pre-microRNAs. Additionally, we aimed to establish a range for evolutionary distance at which differentiation into species can be achieved. We were able to show that discrimination among hominids is fairly impossible while the comparison between, for example, human and worms is straightforward. In the future, pre-miRNA classification strategy which can assign an unknown pre-miRNA to the most likely species of origin may be developed, which will be important in studies depending on deep sequencing data which often contain contaminating sequences [30].

## Methods

### Datasets

We downloaded microRNAs from three different clades (Hominidae, Nematoda, and Pisces) available on miRBase (Release 21); for details see Table 1.

**Table 1:** List of the species whose miRNAs were used in the present study and their amounts available on miRBase. The number next to the species grouping (e.g.: Hominidae) indicates the total amount of miRNAs for that group

Species	Number of pre-miRNAs	Species	Number of pre-miRNAs	Species	Number of pre-miRNAs
Hominidae	3629	Nematoda	1856	Pisces	1623
*Gorilla gorilla*	352	*Ascaris suum*	97	*Cyprinus carpio*	134
*Homo sapiens*	1881	*Brugia malayi*	115	*Danio rerio*	346
*Pan paniscus*	88	*Caenorhabditis brenneri*	214	*Fugu rubripes*	131
*Pongo pygmaeus*	642	*Caenorhabditis briggsae*	175	*Hippoglossus hippoglossus*	40
*Pan troglodytes*	655	*Caenorhabditis elegans*	250	*Ictalurus punctatus*	281
*Symphalangus syndactylus*	11	*Caenorhabditis remanei*	157	*Oryzias latipes*	168
		*Haemonchus contortus*	188	*Paralichthys olivaceus*	20
		*Pristionchus pacificus*	354	*Salmo salar*	371
		*Panagrellus redivivus*	200	*Tetraodon nigroviridis*	132
		*Strongyloides ratti*	106		

Pre-miRNAs in Table 1 were filtered according to sequence similarity on a per species basis to ensure that there is no bias due to multiple identical pre-miRNAs and for human; for example, from the initial 1881 available pre-miRNAs 121 were filtered leaving 1760 for machine learning.

In addition to the main data used in this study (Table 1), we used several clades from miRBase and during those experiments; all pre-miRNAs from all species in those clades were combined into one dataset. For example, the Fabaceae dataset consisted of *Acacia auriculiformis*, *Arachis hypogaea*, *Acacia mangium*, *Glycine max*, *Glycine soja*, *Lotus japonicus*, *Medicago truncatula*, *Phaseolus vulgaris*, and *Vigna unguiculata* totaling about 1400 pre-miRNAs.

### Parameterization of pre-miRNAs

#### K-mers

Simple sequence-based features have been described and used for ab initio pre-miRNA detection in numerous studies. These sequence features, also called words, k-mers, or n-grams, describe a short sequence of nucleotides. For example, a 1-mer over the relevant alphabet can produce the words A, U, C, and G; while a 2-mer over {A, U, C, G} can generate: AA, AC, …, and UU. Higher k have also been used [31], but here we chose 1, 2, and 3-mers as features since most previous studies restrict k (<= 3), because longer k are less likely to be exactly conserved among species, and since sequence motifs cover longer sequences as features. For counting their frequency, each k-mer was detected in the input sequences and divided by the number of k-mers in the sequence given by len(sequence) - k + 1. We calculated k-mers with k = {1, 2, 3} resulting in 84 different features per example.

#### Motif features

Motif features are different from *k*-mers in that they are not exact and allow some degree of error-tolerance. Here a sequence motif is a short stretch of nucleotides that is frequent among a set of pre-miRNAs. Motif discovery, in turn, is the process of finding such short sequences within a larger pool of sequences. The MEME (Multiple Expectation Maximization for Motif Elicitation) Suite [32] was used for motif discovery. The algorithm is based on [33] which works by repeatedly searching for ungapped sequence motifs that occur within input sequences. MEME turned out to be the bottleneck in our analysis workflow, causing long processing times for motif extraction. MEME provides the results as regular expressions and sequence profiles. In our previous work, we represented motifs by using the regular expressions provided by MEME [4, 14, 34]. However, regular expressions only allow for equally probable options at each position and, therefore, profiles are more discriminative since they allow frequencies for each nucleotide option at each sequence position. We, thus, chose profiles to calculate motif scores. 100 motifs were discovered using MEME on a per species basis. Thus 200 motif features were calculated for each input sequence; 100 from either species. We chose 100 motifs per class since for some experiments in this work only few examples were available, and choosing more than 100 motifs would have led to few sequences supporting each discovered motif. 100 motifs mean, that on average (considering all experiments in this study) we expect ten examples to support each motif. For calculation, profiles were aligned with the target sequence and shifted along until the end of the profile reached the end of the sequence or vice versa in case the profile is longer than the sequence. At each position, a score was calculated by adding up the frequencies in the profile for matching nucleotides at their respective positions. The motif position leading to the highest score was reported as the final score for that input sequence. Motif lengths ranged between 11 and 50 with an average of 38. Among selected motifs (i.e.: passing feature selection; see below), the average length was about 40 (Additional file 1: Table S1). The amount of selected motif features among experiments ranged between 15 and 84% with an average of about 40% motif features among the selected ones (Additional file 1: Table S1, Selected Motifs). The number of selected motif features is strongly impacted by the amount of data available. This impact leads to fewest number of selected motifs for *Gorilla gorilla* (30%) followed by *Homo sapiens* (43%) and most selected motifs for experiments involving Hominidae (51%; Additional file 1: Table S1, Selected Motifs).

#### Feature vector and feature selection

Each example is described by 84 k-mer and 200 motif features. However, not all features are equally efficient in separating between positive and negative class. Since information gain has previously been used for feature selection [35], we used KNIME (version 3.1.2) [36] to calculate information gain on a per experiment basis. The 100 features with highest information gain were accepted as the feature set used during model establishment to select from the possible features in the present study:

A, C, G, U, AA, AC, AG, AU, CA, CC, CG, CU, GA, GC, GG, GU, UA, UC, UG, UU, AAA, AAC, AAG, AAU, ACA, ACC, ACG, ACU, AGA, AGC, AGG, AGU, AUA, AUC, AUG, AUU, CAA, CAC, CAG, CAU, CCA, CCC, CCG, CCU, CGA, CGC, CGG, CGU, CUA, CUC, CUG, CUU, GAA, GAC, GAG, GAU, GCA, GCC, GCG, GCU, GGA, GGC, GGG, GGU, GUA, GUC, GUG, GUU, UAA, UAC, UAG, UAU, UCG, UCU, UCA, UCU, UGA, UGC, UGG, UGU, UUA, UUC, UUG, UUU, Motif1, Motif2, Motif3, …, Motifn; where n = 200.

Information gain as available in KNIME is implemented according to Yang and Pedersen [37]. It describes the goodness of a term and in this case how well a feature separates between the positive and negative class compared to other available features. We have previously shown that 50 features may be enough to establish successful models [12] but chose to be conservative here and used 100 features. Additional file 2: Figure S6 shows the impact of number of features for test data and holdout data for this study.

### Classification approach

**Fig. 1:**
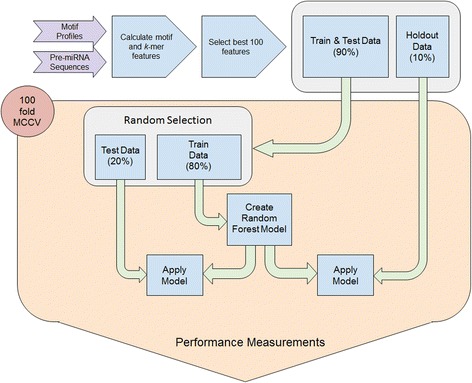
Workflow for model establishment. Data is transformed into a feature vector, and the best 100 features are selected. Initially, 10% data is withheld from the 100-fold MCCV training and testing scheme. All performance measures for testing and holdout data are collected during CV and reported at the end of the workflow

Initially, we performed tests using support vector machines [38], decision trees (DT), Naive Bayes (NB), and random forest (RF) classifiers, but since RF generally outperformed the other methods, we only used RF for the remainder of the study. All classifiers used are part of the data analytics platform KNIME [36], and we used that platform for all analyses. The classifiers were trained and tested using the following parameters. Initially, 10% of the examples were set aside as holdout data, and the remaining 90% of the data were split into 80% training and 20% testing data. Negative and positive examples were forced to equal amounts since we showed that that is important for the successful model establishment in pre-miRNA detection [12]. 100-fold Monte Carlo cross-validation [39] was used to establish the model, and its performance was recorded for each fold. Additionally, for each fold performance was tested on the holdout dataset (Fig. 1). Feature selection is computationally expensive [40] and was, therefore, done before training the models. Additionally, we tested the difference when performing feature selection in each cross-validation iteration (24) for one example (Hominidae vs. Laurasiatheria). We found that features generally achieved similar ranks for the 24 iterations (Additional file 1: Table S1; Additional file 2: Figure S1). Additionally, we observed that there was no relevant impact on the accuracy distribution for the 24 tests (Additional file 2: Figure S2). Therefore, we used the model establishment schema as described in Fig. 1.

#### Performance evaluation

For each established model we calculated a number of performance measures like the Matthews correlation coefficient (MCC) for the evaluation of the classifier such as sensitivity, specificity and accuracy according to the following formulations (with TP: true positive, FP: false positive, TN: true negative, and FN referring to false negative classifications): [41]

$$ \begin{array}{l}\mathrm{Sensitivity} = \mathrm{TP}/\left(\mathrm{TP} + \mathrm{F}\mathrm{N}\right);\ \mathrm{SE},\ \mathrm{Recall}\\ {}\mathrm{Specificity} = \mathrm{TN}/\left(\mathrm{TN} + \mathrm{F}\mathrm{P}\right);\ \mathrm{SP}\\ {}\mathrm{P}\mathrm{recision} = \mathrm{TP}/\left(\mathrm{TP} + \mathrm{F}\mathrm{P}\right)\\ {}\mathrm{F}\hbox{-} \mathrm{Measure} = 2\ *\left(\mathrm{precision}\ *\ \mathrm{recall}\right)/\left(\mathrm{precision} + \mathrm{recall}\right)\\ {}\mathrm{Accuracy} = \left(\mathrm{TP} + \mathrm{TN}\right)/\left(\mathrm{TP} + \mathrm{TN} + \mathrm{F}\mathrm{P} + \mathrm{F}\mathrm{N}\right);\ \mathrm{ACC}\\ {}\mathrm{MCC}\kern0.5em =\frac{\left(\mathrm{TP}\backslash\ \mathrm{TN}\hbox{-} \mathrm{F}\mathrm{P}\backslash\ \mathrm{F}\mathrm{N}\right)}{\sqrt{\left(\mathrm{TP}+\mathrm{FP}\right)\left(\mathrm{TP}+\mathrm{FN}\right)\left(\mathrm{TN}+\mathrm{FN}\right)\left(\mathrm{TN}+\mathrm{FP}\right)}};\end{array} $$

All reported performance measures refer to the average of 100-fold Monte Carlo Cross Validation (MCCV). Since single statistics (e.g.: averages) are of limited value to describe machine learned models, and since receiver operator characteristic curves for hundreds of trained models would be hard to assess, we calculated accuracy distribution for all models trained and used them to describe model performance.

## Results and discussion

The random forest (RF) classifier was used to establish machine learned models using a 10/80/20 split for holdout, training, and testing, respectively. 100-fold MCCV was used to train, test, and apply models to constant holdout data. The number of pre-miRNA examples available on miRBase per species is quite variable and to ensure similar numbers of positive and negative examples, groups of species had to be considered. One such group is Hominidae which consists of human and the great apes. Specifically, *Homo sapiens*, *Gorilla gorilla*, *Pan paniscus*, *Pongo pygmaeus*, *Pan troglodytes*, and *Symphalangus syndactylus* have available pre-miRNA examples in miRBase (Table 1). Taking Hominidae as positive data and pre-miRNAs from various other groups as negative data models to differentiate the groups were trained and their performance established (Table 2).

**Table 2:** Average performance of models trained to classify into hominidae or one of the listed clades. The best 100 features were selected based on information gain and training/testing was performed with a 10/80/20 split at 100-fold MCCV

Hominidae vs.	Holdout			Test		
	F-measure	Accuracy	MCC	F-measure	Accuracy	MCC
Hexapoda	0.93	0.93	0.86	0.93	0.93	0.86
Brassicaceae	0.82	0.93	0.78	0.92	0.92	0.84
Monocotyle	0.88	0.92	0.83	0.91	0.91	0.82
Nematoda	0.87	0.91	0.80	0.90	0.90	0.80
Fabaceae	0.81	0.88	0.72	0.87	0.87	0.75
Pisces	0.80	0.86	0.70	0.86	0.86	0.72
Virus	0.44	0.83	0.43	0.82	0.82	0.64
Aves	0.59	0.75	0.41	0.72	0.72	0.45
Laurasiatheria	0.54	0.73	0.39	0.70	0.72	0.45
Rodentia	0.62	0.69	0.37	0.69	0.69	0.38
*Homo sapiens*	0.62	0.61	0.23	0.62	0.61	0.23
Cercopithecidae	0.26	0.51	0.01	0.50	0.50	0.01

Note, that for the test Hominidae vs *H. sapiens* the *H. sapiens* examples were removed from Hominidae. Table is sorted according to average model accuracy. This table presents average accuracy values, but Additional file 2: Figures S3-S5 present the accuracy distributions for 100 fold MCCV

**Fig. 2:**
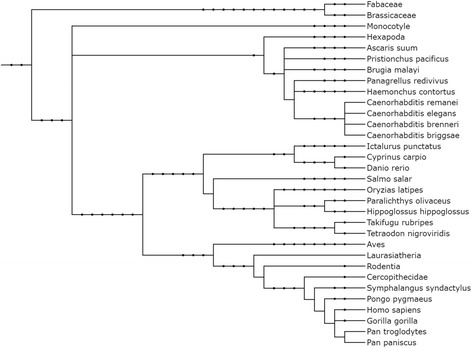
Phylogenetic relationship among organisms and groups used in the present study (excluding viruses). Itol (http://itol2.embl.de/) was used to create the phylogenetic tree [42]. Newick and PhyloXML formatted files to build the tree are available as Additional files 3 and 4: Files S2 and S3, respectively

Performance on holdout data is very similar to the testing performance (Table 2). Classifying into Hominidae or Hexapoda was very accurate (0.93 accuracy) while classification into Hominidae or Cercopithecidae was impossible (0.50 accuracy) which is likely due to the very close evolutionary relationship (Fig. 2). To assess this further, the human pre-miRNA examples were removed from the Hominidae dataset. This data was used to establish a model versus human. A slightly better accuracy of 0.61 compared to Hominidae vs. Cercopithecidae was achieved. Since in Hominidae about half of the pre-miRNA examples stem from human and the evolutionary distance is also very low, a similar result to the one of Hominidae vs. Cercopithecidae was expected.

Results in Table 2 and phylogenetic relationship among organisms and groups used in the present study (Fig. 2) show a similar trend. Organisms closely related also show similar average model accuracy, and with increasing phylogenetic distance the average model accuracy also increases in general.

**Fig. 3:**
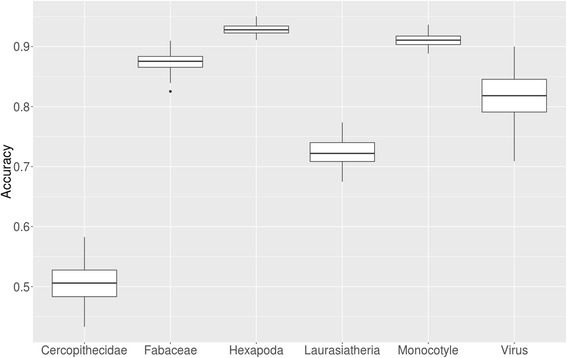
Accuracy distribution over 100-fold MCCV for six selected species and groups of species against Hominidae

Since an average accuracy can be misleading, the accuracy distribution over 100-fold MCCV during machine learning was reported (Fig. 3). The interquartile ranges summarizing the 100 fold MCCV model training were quite small and only slightly increased with lower average accuracy. Thereby, confirming that training models was successful on average and not based on outliers or overfitting. Only few virus examples (<300) are available on miRBase and those targeting human also need similar sequences to human miRNAs. On the other hand, those targeting the viruses themselves should not have similar sequences to human. Therefore, the interquartile range is larger for viruses and the overall accuracy distribution is lower than for other examples.

*Gorrilla gorilla*, also in the hominidae group, has a sufficient amount of pre-miRNA examples to establish a model and, therefore, for human and gorilla versus other species and groups of organisms models were trained in parallel for comparison (Table 3). Since human and gorilla are very closely related, they should show similar average model accuracies when trained against the same species.

**Table 3:** Average accuracy (ACC) and Matthews correlation coefficient (MCC) for 100-fold MCCV model training using *Homo sapiens* (HSA) or *Gorilla gorilla* (GGO) as target class and Nematoda or Pisces as other class (sorted by HSA ACC). Results for HSA and GGO vs all Nematoda and Pisces are bolded

	Versus	HSA ACC	GGO ACC	HSA MCC	GGO MCC
Nematoda	*Caenorhabditis brenneri*	0.94	0.96	0.88	0.93
	*Pristionchus pacificus*	0.93	0.94	0.86	0.88
	*Panagrellus redivivus*	0.93	0.96	0.86	0.92
	*Strongyloides ratti*	0.91	0.95	0.82	0.90
	*Caenorhabditis remanei*	0.89	0.87	0.78	0.75
	*Caenorhabditis briggsae*	0.87	0.86	0.75	0.72
	*Ascaris suum*	0.86	0.87	0.73	0.75
	*Haemonchus contortus*	0.86	0.87	0.72	0.75
	*Caenorhabditis elegans*	0.86	0.87	0.71	0.73
	*Brugia malayi*	0.84	0.80	0.68	0.60
	*Nematoda*	0.89	0.88	0.78	0.68
Pisces	*Salmo salar*	0.92	0.97	0.84	0.94
	*Ictalurus punctatus*	0.89	0.96	0.78	0.92
	*Paralichthys olivaceus*	0.84	0.93	0.71	0.87
	*Oryzias latipes*	0.83	0.77	0.67	0.56
	*Danio rerio*	0.80	0.78	0.60	0.56
	*Fugu rubripes*	0.77	0.79	0.55	0.59
	*Cyprinus carpio*	0.76	0.77	0.53	0.53
	*Tetraodon nigroviridis*	0.76	0.79	0.53	0.58
	*Hippoglossus hippoglossus*	0.67	0.69	0.35	0.39
	*Pisces*	0.84	0.83	0.68	0.57

Nematoda are evolutionary distant from Hominidae and it was our expectation to create well-performing models. In general, that expectation correlates with the results and all models achieve more than 80% average accuracy. However, there is a trend towards species with more examples on miRBase to create models which better discriminate between species. More examples generally lead to better models and this finding is just a confirmation of that concept. *C. elegans* is an outlier in this respect since it has second most examples on miRBase which indicates that some of those reported pre-miRNAs may not actually be miRNAs. Pisces is evolutionarily closer to human than Nematoda but still distant and, therefore, we expected models with slightly lower performance. In general, this expectation held true although *H. hippoglossus* performed particularly bad which is likely due to the low amount of examples (40) some of which may additionally be wrong. Interestingly, the fish with lowest number of examples, *P. olivaceus* (20), performed quite well which is likely due to the calculation of performance measures which may return biased results for classes with very few members. It may additionally mean that the reported miRNAs are of high quality. Human and gorilla results are very similar and confirm that the results are not by chance. Furthermore, when training human or gorilla against the complete group of Pisces or Nematoda, results similar to the expected group average are obtained which shows that actual behavior is consistent with the expectation.

### Motif construction could cause spurious results

In order to ensure, that the results are not due to improper motif selection or due to chance, we performed an experiment with 10-fold MCCV where motifs were extracted from randomly chosen 50% of the input data in each fold. For this experiment, we selected Hominidae versus Laurasiatheria since they represented average performance compared to all other tested models (Table 2). In each fold, 10-fold MCCV was used to establish RF models which lead to a total of 100 RF models.

**Fig. 4:**
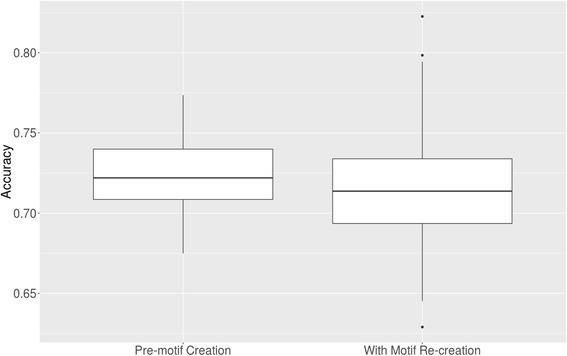
Model accuracy distribution for models trained with pre-created motifs and for the workflow where motifs were created in each iteration

As expected, the average classification performance (0.71) overall 100-folds was similar to the previous performance (0.72), indicating that feature calculation and extraction were performed properly. Not only was the average performance very similar also the accuracy distribution for these two experiments was (Fig. 4).

The interquartile range for the pre-created motifs was somewhat smaller than for the motif re-creation approach (Fig. 4), but that can be expected since only 50% of the data was used for motif finding which should lead to lower quality motifs. Additionally, the average accuracy for the second approach was about a percent lower, but in general, the distributions are similar. Finally, motif re-creation per MCCV fold introduces more outliers which are likely due to overfitting. Therefore, motifs should be discovered using the entire dataset, and they should not be recreated using a subset of the data in each training iteration.

## Conclusions

Machine learning has become an important part of pre-miRNA detection, but it suffers from missing *bona fide* negative data [8]. The current aim in the field is to detect pre-miRNAs in, for example, genomes. A previous classification of pre-miRNAs into groups has also been performed and detected conserved miRNA families [9]. On the other hand, it has been shown that miRNAs can evolve rapidly [10–12]. Therefore, we were interested in whether a machine learned model could be trained to classify miRNAs based on their species of origin. To achieve this, we used one species’ pre-miRNAs as positive and the other’s pre-miRNAs as negative data for the establishment of models. The features we employ are all sequence-based since structural features should be more conserved thereby concealing smaller evolutionary distances.

We showed that sequence motifs and *k*-mer features were properly created (Fig. 4). In the same way, a model was established for Hominidae versus selected clades available on miRBase, and the average accuracy closely mirrored the evolutionary distance (Table 2; Fig. 2). To check this result, human and gorilla were used as target species and trained against Nematoda and Pisces species available on miRBase. Both targets lead to comparable results (Table 3), thereby confirming the viability of this approach. In conclusion, we show that a classifier can differentiate between pre-microRNAs from different species using a combined motif and *k*-mer signature. In future studies, this may lead to the ability to classify unknown pre-miRNAs into their correct category which is important when attempting studies involving xeno-miRNAs in order to separate interesting results from contamination. To achieve that end, models for all known pairs of species need to be established. Applying all models to an unknown example then creates a fingerprint for that example. After that multi-class classification or clustering (self-organizing maps, nearest neighbor, etc.) can be applied to determine class/cluster membership of the unknown example. This approach would use the distance information of all trained species models and would be much more powerful than applying multi-class classification or clustering directly to the examples. Thereby, unknown examples can be assigned a species of origin using a fingerprint.

## Additional files

Additional file 1: Table S1. Extracted Motifs: All of the extracted sequence motifs are listed, as well as information gain scores for 24-fold cross validation. (XLSX 345 kb)
Additional file 2: Figures S1 to S6:
**Figure S1** shows the rank distribution for k-mers and motif features. **Figure S2** displays how feature selection impacts accuracy. **Figures S3** and **S4** provide additional accuracy distributions for various clades versus hominidae and **Figure S5** provides similar information for Cercopitheciadae and Hominidae versus human. Figure S6 supports the choice of selected number of features. (DOCX 653 kb)
Additional file 3: File S2. Newick formatted phylogenetic tree: This file can be directly uploaded to Itol or other phylogenetic tree viewers for further analysis. (TXT 2 kb)
Additional file 4: File S3. PhyloXML formatted phylogenetic tree: This file can be directly uploaded to Itol or other phylogenetic tree viewers for further analysis. (TXT 7 kb)

## Abbreviations

ACC: Accuracy
DT: Decision tree
FN: False negative
FP: False positive
MCC: Matthews correlation coefficient
MCCV: Monte Carlo cross validation
MEME: Multiple expectation maximization for motif elicitation
miRNA: microRNA
NB: Naïve Bayes
RF: Random forest
RISC: RNA-induced silencing complex
TN: True negative
TP: True positive
