# Supplementary material for: MicroRNA categorization using sequence motifs and k-mers
Source: BMC Bioinformatics. 2017 Mar 14;18:170. doi: 10.1186/s12859-017-1584-1 (PMC5351198; doi:10.1186/s12859-017-1584-1)
Supplement: Additional file 2: Figures S1 to S6: — Figure S1 shows the rank distribution for k-mers and motif features. Figure S2 displays how feature selection impacts accuracy. Figures S3 and S4 provide additional accuracy distributions for various clades versus hominidae and Figure S5 provides similar information for Cercopitheciadae and Hominidae versus human. Figure S6 supports the choice of selected number of features. (DOCX 653 kb) [file 12859_2017_1584_MOESM2_ESM.docx]

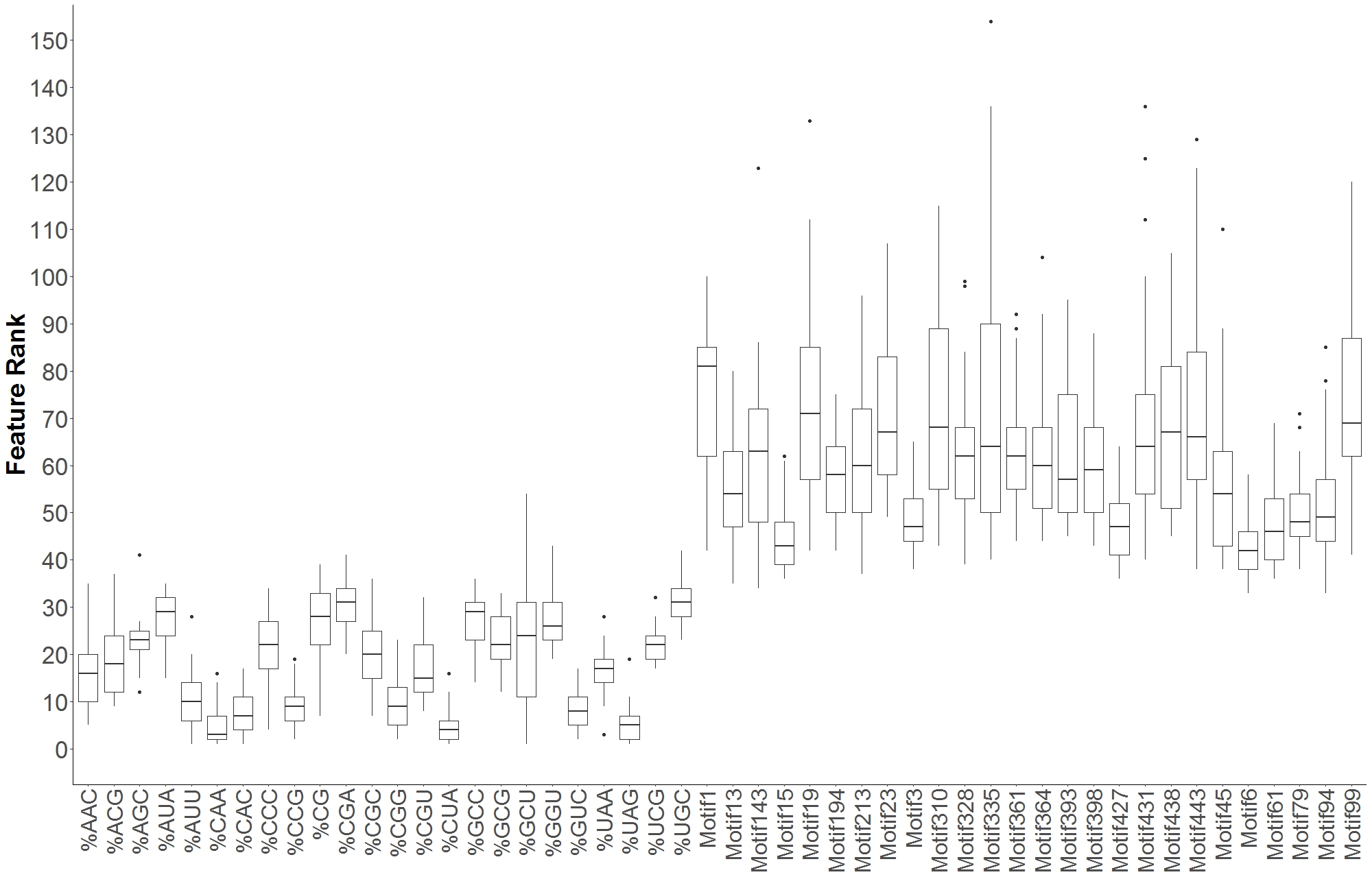


Figure S1: Rank distribution for features over 24 rounds of cross validation (CV). Motifs were discovered for 50% of the available data and in each round of CV features were ranked using information gain. The ranked features are available in Table S1 (Motifs24CV). Ranks for all features for all folds are also available in Table S1 (Ranked Features).


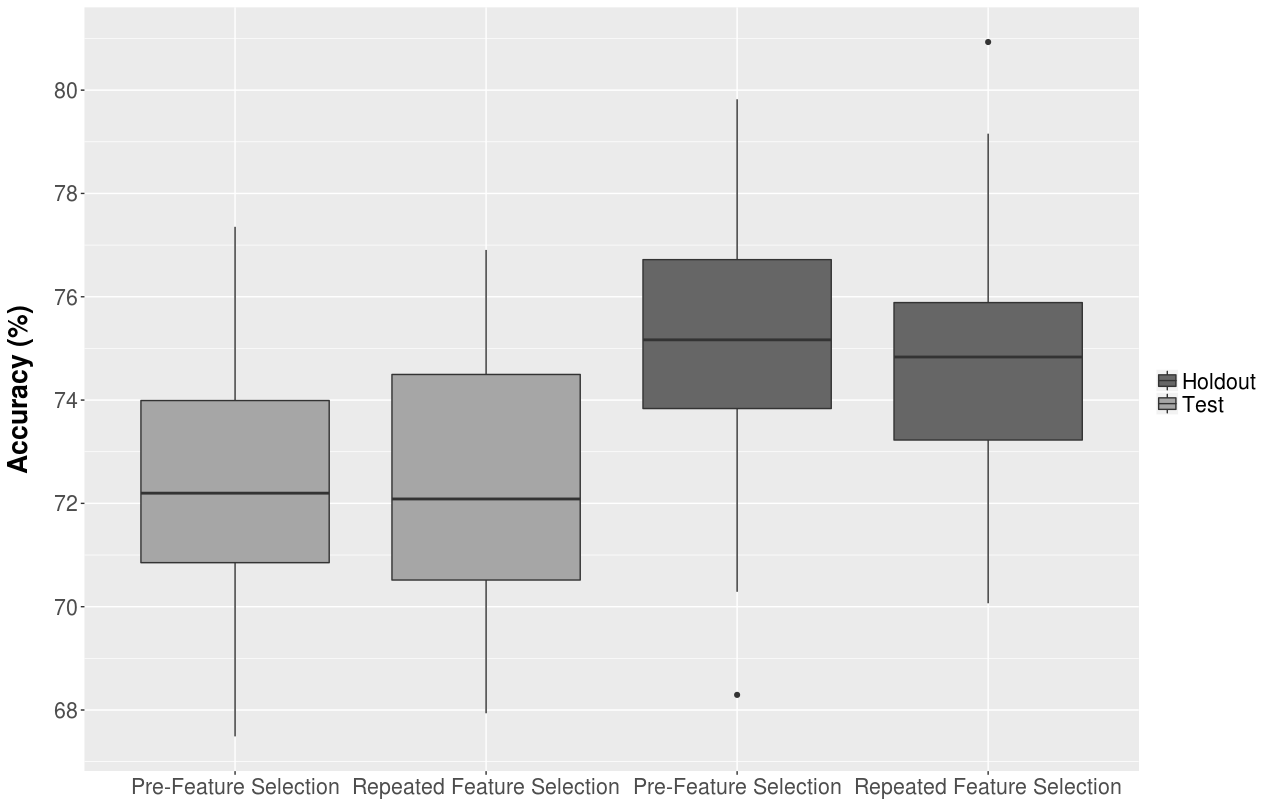


Figure S2: Accuracy distribution for feature selection prior to training and testing and using a strategy where feature selection is repeated for each training-testing iteration.


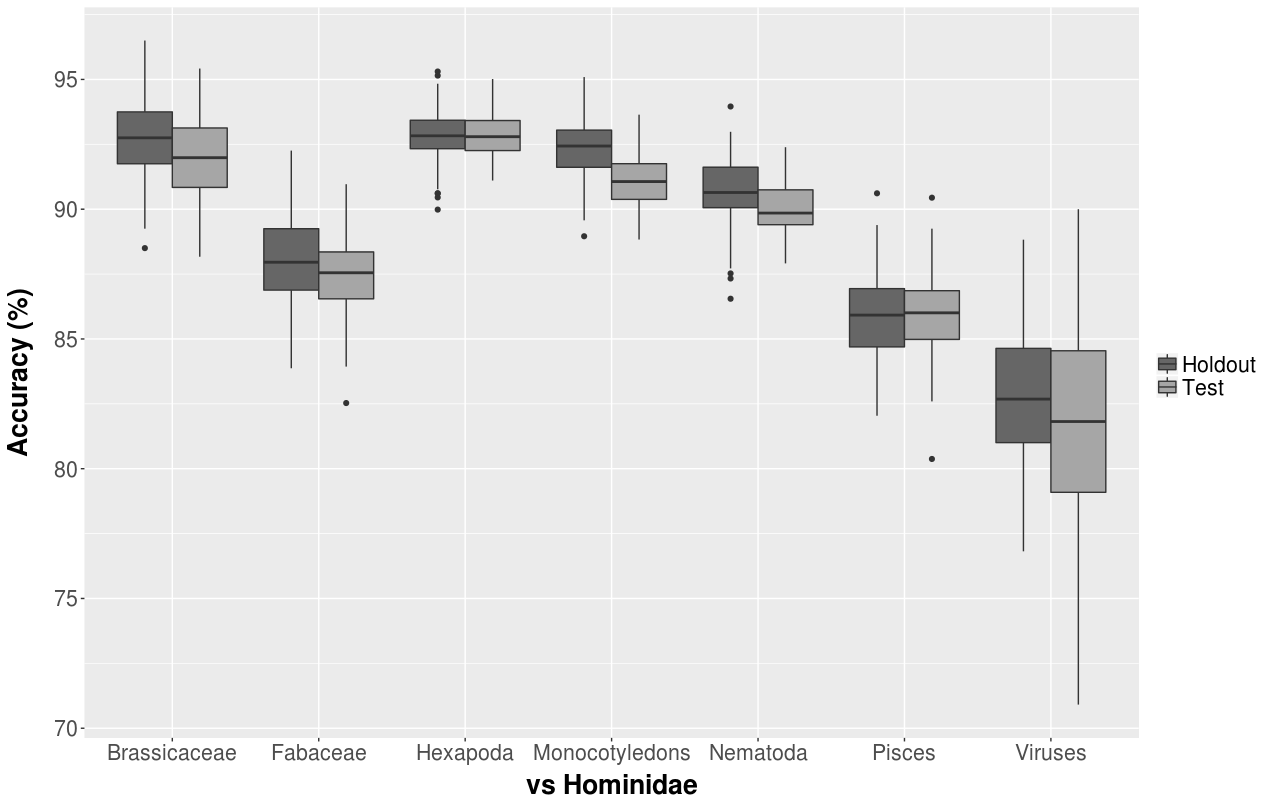


Figure S3: Accuracy distribution for 100 fold MCCV for selected species (and groups) against hominidae.


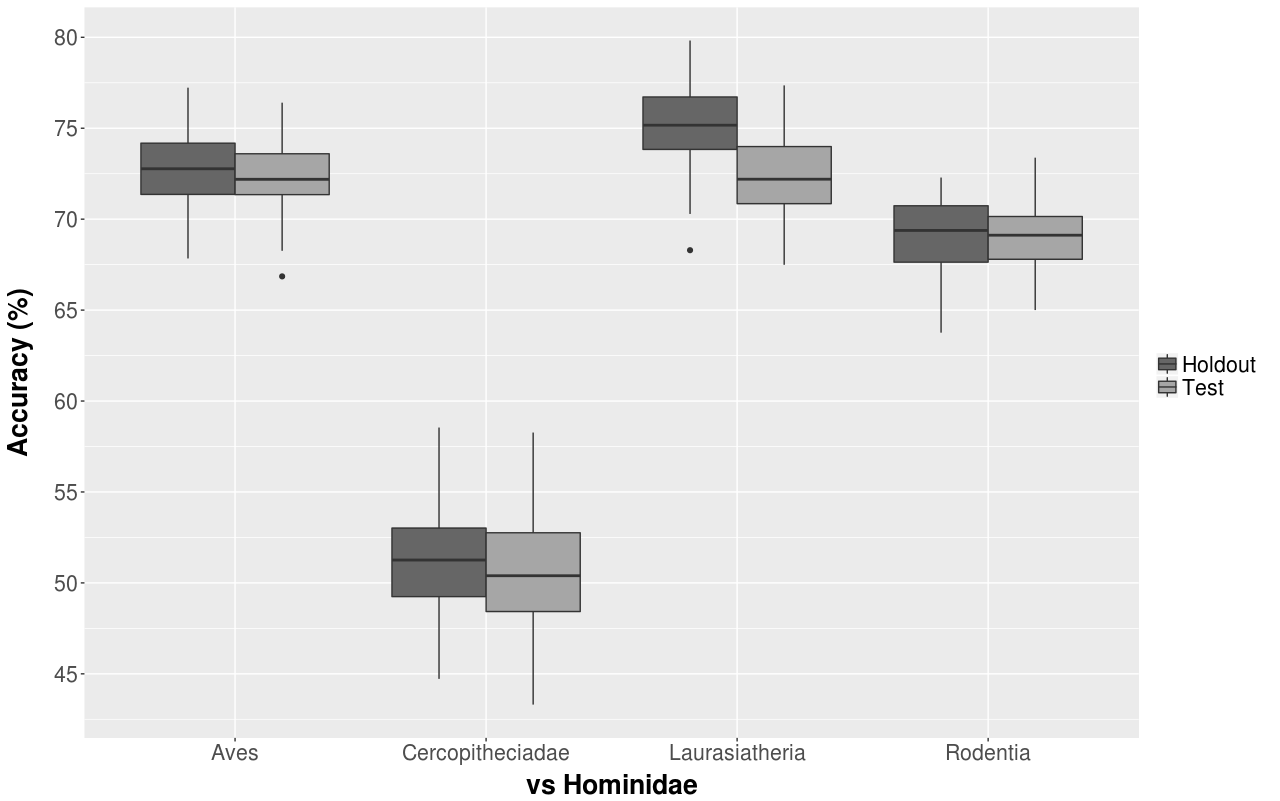


Figure S4: Accuracy distribution for 100 fold MCCV for selected species (and groups) against hominidae.


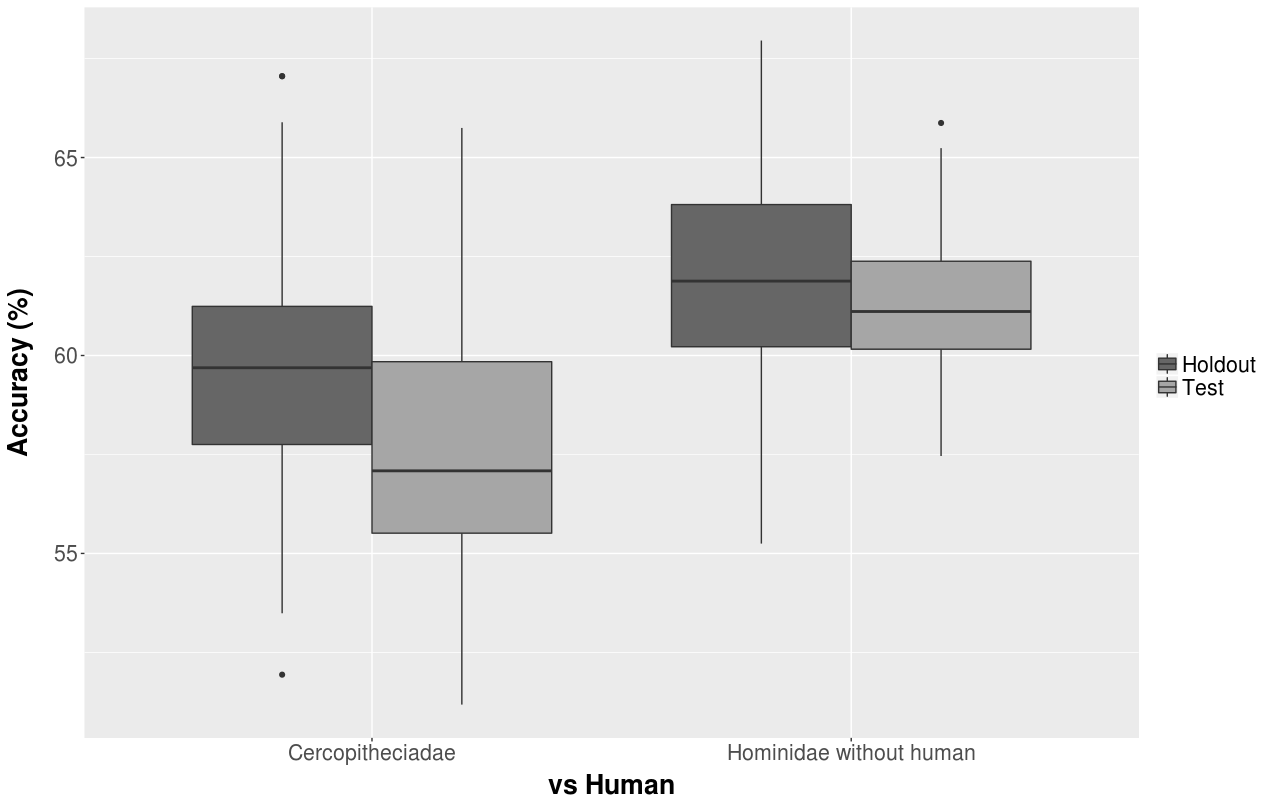


Figure S5: Accuracy distribution for 100 fold MCCV for Cercopitheciadae and hominidae against human.


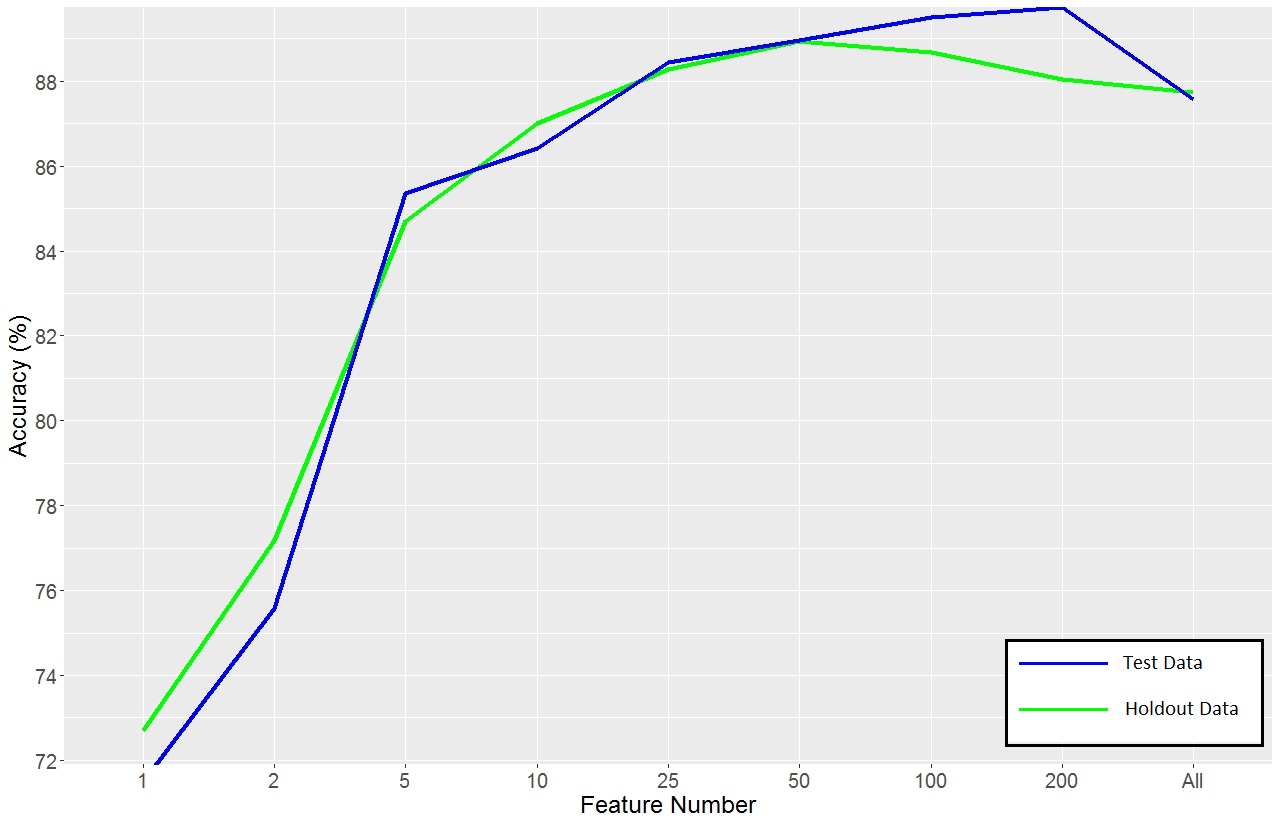


Figure S6: Features were sorted by information gain (decreasingly). Feature sets from 1 to all features were created incorporating features with higher information gain first. Then models were created for each feature set and were tested on test and holdout data. The results are presented. For holdout data more than 100 features shouldn’t be used while for test data 100 features almost presents the best selection.
